# Advances in stem cell therapy for peritoneal fibrosis: from mechanisms to therapeutics

**DOI:** 10.1186/s13287-023-03520-3

**Published:** 2023-10-10

**Authors:** Weiyan Huang, Demeng Xia, Wendi Bi, Xueli Lai, Bing Yu, Wei Chen

**Affiliations:** 1 Department of Nephrology, Shanghai Changhai Hospital, Naval Medical University, Shanghai, China; 2 Department of Pharmacy, Seventh People’s Hospital of Shanghai University of Traditional Chinese Medicine, Shanghai, China; 3 Department of Cell Biology, Center for Stem Cell and Medicine, Naval Medical University (Second Military Medical University), Shanghai, China

**Keywords:** Stem cell, Peritoneal fibrosis, Mesothelial–mesenchymal transition, Mechanism, Cell therapy

## Abstract

Peritoneal fibrosis (PF) is a pathophysiological condition caused by a variety of pathogenic factors. The most important features of PF are mesothelial–mesenchymal transition and accumulation of activated (myo-)fibroblasts, which hinder effective treatment; thus, it is critical to identify other practical approaches. Recently, stem cell (SC) therapy has been indicated to be a potential strategy for this disease. Increasing evidence suggests that many kinds of SCs alleviate PF mainly by differentiating into mesothelial cells; secreting cytokines and extracellular vesicles; or modulating immune cells, particularly macrophages. However, there are relatively few articles summarizing research in this direction. In this review, we summarize the risk factors for PF and discuss the therapeutic roles of SCs from different sources. In addition, we outline effective approaches and potential mechanisms of SC therapy for PF. We hope that our review of articles in this area will provide further inspiration for research on the use of SCs in PF treatment.

## Introduction

Peritoneal fibrosis (PF) is a common peritoneal lesion caused by numerous pathological conditions in clinical practice. For instance, progressive fibrosis may eventually develop in 50% of peritoneal dialysis (PD) patients. Currently, PD accounts for approximately 11% of all forms of life-sustaining renal replacement therapy worldwide [1, 2], and chronic PF-related ultrafiltration failure has become a significant factor in PD patient dropout rates. Furthermore, chronic stomach pain, female infertility and mechanical intestinal obstruction can be caused by peritoneal adhesions due to acute postoperative PF. These factors bring tremendous pain and stress to the patient as well as a huge economic burden to society.

PF is characterized by peritoneal mesothelial cell (PMC) loss, angiogenesis, increased myofibroblast numbers and abnormal production of extracellular matrix (ECM) proteins, resulting in progressive thickening of the peritoneal membrane (PM) submesothelial compact zone [3]. During this process, mesothelial–mesenchymal transition (MMT) has been shown to play a significant role in the induction of fibrosis and the subsequent functional deterioration of the PM. Current treatments to minimize this process mainly include improving PD catheter placement techniques, improving the biocompatibility of peritoneal dialysis solution (PDS) and using pharmacological agents such as tamoxifen, which has been used to alleviate peritoneal inflammation and fibrosis [4, 5]. However, these approaches may have limitations and unfavourable side effects, such as gastrointestinal reactions to drugs, and they are only partially effective. Thus, it is crucial to conduct research on other methods of PF prevention, such as cell-based therapy.

In recent years, research on stem cells (SCs) has rapidly increased to support the use of SCs in clinical applications. SCs are biological units that are responsible for the development and regeneration of organ and tissue systems, and they have received much attention in the fields of differentiation, transplantation and the immune response in the contexts of various diseases [6]. Many studies have shown that SCs can alleviate PF in vivo and in vitro, and relevant clinical trials have also been reported, with mesenchymal SCs (MSCs) being the most extensively studied. However, the function and mechanism of SCs in PF have not been thoroughly summarized. In this review, we discuss the risk factors for PF and provide a brief overview of the therapeutic potential of available SCs for this disease process.

## Risk factors for peritoneal fibrosis

The peritoneum is a serosal membrane that forms the lining of the abdominal cavity. It is made up of a continuous monolayer of mesodermal-derived cells called mesothelial cells (MCs). MCs are epithelial-like cells that are supported by a submesothelial region composed of a thin layer of connective tissue containing lymphatics, blood vessels, resident inflammatory cells and fibroblast-like cells [7]. The development of PF is attributed mainly to increased accumulation of activated myofibroblasts. It has been well demonstrated that active (myo-)fibroblasts, which are stimulated by a variety of fibrogenic cytokines in the injured environment, are the dominant collagen-producing cells during organ fibrosis [8], generating an excessive amount of ECM and leading to the destruction of normal tissue architecture. The sources of active fibroblasts are varied and can include local interstitial fibroblasts, bone marrow-derived circulating fibrocytes, pericytes (or perivascular fibroblasts), endothelial cells, local mesenchymal progenitor cells and pathological MCs, which produce active fibroblasts via a process known as MMT [9–11].

It is thought that MMT starts in the context of organ fibrosis as a component of a repair process that typically produces fibroblasts and other related cells to rebuild tissues after injury and ceases once the stimuli are attenuated. However, MMT can pathologically continue and react to stimuli, ultimately resulting in tissue structure breakdown and fibrosis. MMT is complicated and involves several processes. (1) During the depolymerization of intercellular junctions, tight junctions (composed of proteins such as zonula occludens-1, ZO-1) and adhesion junctions (composed of proteins such as E-cadherin) between MCs are loosened, while mesenchymal junctional proteins (composed of proteins such as N- and OB-cadherin) are strengthened. The microvilli vanish as the cell loses polarity. (2) Cytoskeletal remodelling and increased expression of α-SMA and FSP1 occur. During MMT, keratin expression is reduced, vimentin expression is increased, and fibroblast-specific protein-1 (FSP1) and α-smooth muscle actin (α-SMA) are resynthesized. (3) Basement membrane disruption promotes cell invasion and migration. The integrity of the basement membrane of MCs is an important structural basis for maintenance of morphology, and disruption of basement membrane integrity triggers MMT [11–13].

**Fig. 1:**
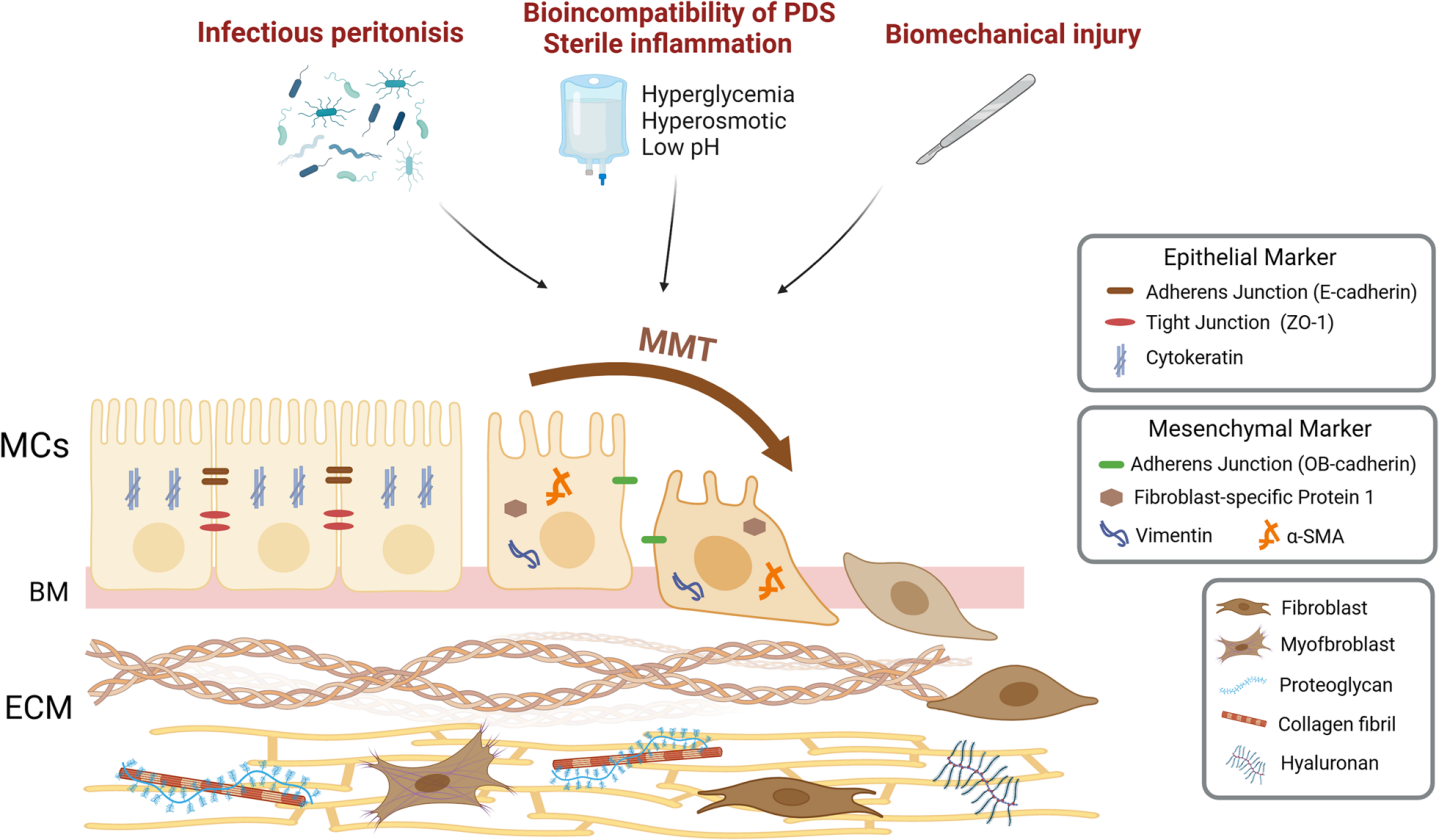
Risk factors that contribute to PF and MMT. Many factors cause MMT, including infectious peritonitis, bioincompatibility of PDS, sterile inflammation and biomechanical injury. *PDS* peritoneal dialysis solution, *MMT* mesothelial–mesenchymal transition, *MCs* mesothelial cells, *BM* basement membrane, *ECM* extracellular matrix, *ZO-1* zonula occludens-1, *α-SMA* α-smooth muscle actin. Created with BioRender.com

There are numerous risk factors that contribute to these changes in the peritoneal mesothelium, which are illustrated in Fig. 1.

### Infectious peritonitis

Peritonitis is the main cause of induced PF. Infectious agents primarily damage the peritoneum via innate pattern recognition receptors (PRRs) on the peritoneum. PRRs recognize pathogens by interacting with molecules that are shared by all microbial species, which are referred to as pathogen-associated molecular patterns (PAMPs) [14].

Infectious peritonitis is the most significant side effect of PD [15]. During PD, indications of fibrosis are discovered in 50 to 80% of patients within one or two years [16]. Gram-positive bacteria that translocate from the skin and Gram-negative bacteria from intestine are responsible for the bulk of peritonitis episodes in PD patients [29]. Fungi also play an important role; while PD-related fungal peritonitis is uncommon, it has the worst prognosis due to the associated severe inflammation [17]. Joel Zindel [18] et al. demonstrated that bacterial contamination triggers mesothelial epidermal growth factor receptor (EGFR) signalling in PF caused by abdominal surgery, which induces MCs to produce myofibroblasts and further causes postsurgical peritoneal adhesion development. In addition, there have been few reports on the roles of viruses, but their effects cannot be ignored.

### Bioincompatibility of PDS and sterile inflammation

The partial bioincompatibility of PDS may stimulate fibrosis, oxidative stress and a microinflammatory state in the peritoneum, leading to progressive morphological and functional changes in the peritoneum. This process includes deterioration of the mesothelium, shedding of MCs and loss of the underlying basement membrane [16, 19].

Traditional PDS features a low pH and a hyperosmotic and hyperglycaemic state. Glucose oxidation may generate harmful glucose degradation products (GDPs), which are highly reactive carbonyls that can be toxic to cells to varying degrees and can impact the proliferation and viability of MCs. In addition, advanced glycosylation end products (AGEs) formed by glucose and reactive carbonyl compounds can attach to free amino groups on proteins or lipids [20] and contribute to peritoneal pathophysiological alterations.

High glucose and its byproducts have been demonstrated to promote MC changes and PF by inducing a microinflammatory state in the peritoneum [21, 22]. These factors can lead to oxidative stress in cells, which can increase the synthesis and reduce the clearance of reactive oxygen species (ROS), resulting in a significant buildup of ROS in living tissue. By recognizing damage-associated molecular patterns (DAMPs) generated by cellular stress, Toll-like receptors (TLRs) mediate sterile inflammation [23]. ROS promote aseptic peritoneal inflammation by triggering TLRs, activating the nuclear factor-κB (NF-κB) signalling pathway [24] and inducing the expression of genes related to inflammation and the secretion of many inflammatory and profibrotic cytokines, such as IL-6, IL-17, IL-1β, transforming growth factor-β1 (TGF-β1) and vascular endothelial growth factor (VEGF) [25]. These factors have cytotoxic effects on MCs, causing peritoneal mesothelial exfoliation and decreases in intercellular junctional protein levels, resulting in peritoneal hyperpermeability [26].

It has recently been shown [27] that high-glucose PDS (HGPDS) can cause MC apoptosis and autophagy. This stimulation also promotes MMT and fibrosis, which leads to matrix deposition, thickening of the submesothelial space and increased vascular density, thus increasing permeability and ultimately resulting in PF [28].

### Biomechanical injury

A substantial body of evidence has shown that, in addition to external biochemical mediators, biomechanical stresses can affect cellular physiopathological reactions [29]. Changes in the biomechanical properties of the ECM, such as changes in ECM stiffness, can alter cell states that are important drivers of the fibrotic response [30].

The PM is constantly exposed to biomechanical stimuli while exposed to PDS. Injection of significant volumes of PDS is necessary for PD. Enlarging the abdominal cavity and mechanically stretching MCs results in mechanical stress. Following abdominal laparotomy, the PM may sustain further mechanical perturbations [18, 31]. Recent research has shown that subjecting MCs to linear cyclic stretch in vitro causes numerous cellular alterations that are consistent with MMT induction. A model that incorporates these experimental results has predicted that MMT can be induced when biomechanical and metabolic signals interact [32].

## Sources of stem cells and new treatments

**Fig. 2:**
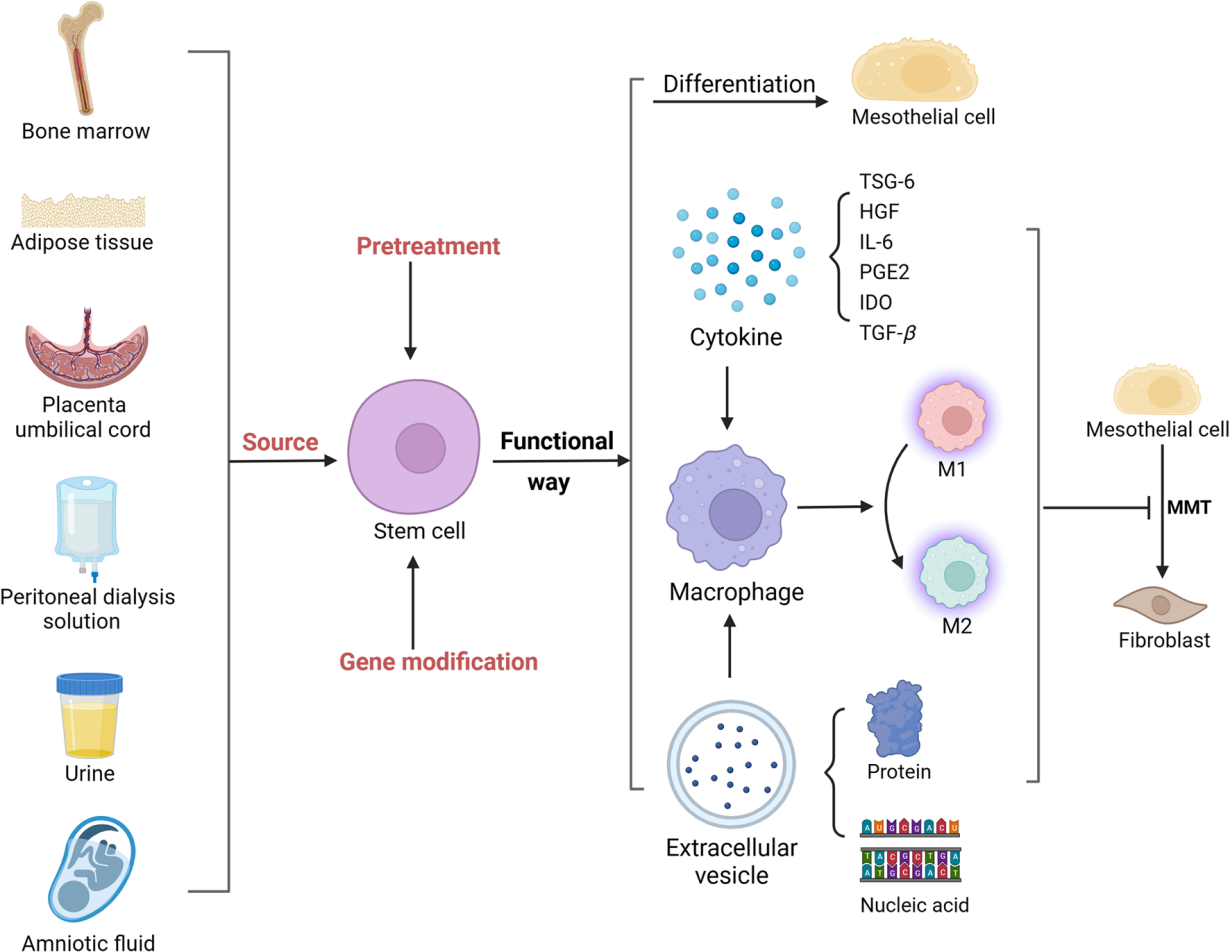
Sources of SCs, novel treatments using SCs and potential mechanisms of SC-based therapy in PF. There are many different sources of SCs, such as bone marrow, adipose tissue, umbilical cord, PDS, urine and amniotic fluid. Pretreatment and genetic modification can also be used to enhance the effects of SCs. SCs act through multiple mechanisms, including by affecting the differentiation of MCs, exerting paracrine effects and regulating inflammatory responses. *MMT* mesothelial–mesenchymal transition. Created with BioRender.com

SCs contain many types and can be isolated from a wide variety of tissues [33], and are suitable for experimental and potentially clinical applications. Many studies have shown that SCs are effective treatments for animal models of PF, and clinical trials have been attempted. The sources of SCs and novel treatments for SCs are summarized in Fig. 2. We also list the SC types that have received the most attention in terms of treatment and their associated studies in Table 1.

**Table 1:** Major stem cell types used for therapy and possible mechanisms

Types of SCs	Possible mechanisms	Examples
MSCs	Settlement and differentiation	BM-MSCs can differentiate into MCs that participate in peritoneal repair and remodelling [34]
	Secretion of cytokines acts on MCs	AD-MSCs release HGF and reduce PF by promoting the migration and proliferation of MCs [35] The secretome and soluble factors from pMSCs have shown a significant decrease in PMC death when exposed to PDS [36]
	Secretion of EVs	Exosomes from BM-MSCs carry a series of miRNAs with different functions and effectively prevent PF [37] EVs from AD-MSCs can induce rat PMC proliferation and migration via stimulation of the MAPK–ERK1/2 and PI3K–AKT axes [38] Exosomal lncRNA GAS5 derived from UC-MSCs can alleviate MMT through the Wnt/β-catenin signalling pathway [39]
	Immunoregulation	BM-MSCs secrete IL-6, IDO and exosomes that affect M2 macrophage differentiation [40–42] AD-MSCs attenuate PF by modulating macrophage polarization via IL-6 [43] Preconditioning of MSCs with serum-free culture conditions, IL-1β, IFN-γ, *Leishmania major* soluble antigens and melatonin can promote anti-inflammatory properties in peritoneal macrophages and reduce PF [40, 44–46] SIRT1-modified MSCs can attenuate inflammation and PF through the TGF-β/Smad3 pathway [47]
USCs	Secretion of EVs	Exosomes from USCs alleviate tissue fibrosis via regulating miR-301b-3p/TGF-βR1 pathway [48]
	Immunoregulation	USCs downregulate the Th1/Th17 immune responses in a PGE2-dependent manner and reduce inflammation [49]
HSCs	Settlement and differentiation	HSCs that originate from the bone marrow have the ability to transform into MCs for repairing and remodelling the peritoneum [34]
AFSCs	Immunoregulation	Paracrine factors produced by AFSCs cause M1-M2 macrophage polarization in a cell contact-independent way and exhibit anti-inflammatory properties [50]

A brief list of the major stem cell types used for therapy and their possible mechanisms, as well as some examples of relevant studies

*SCs* stem cells, *MSCs* mesenchymal stem cells, *BM-MSCs* bone marrow MSCs, *MCs* mesothelial cells, *AD-MSCs* adipose-derived MSCs, *HGF* hepatocyte growth factor, *PF* peritoneal fibrosis, *PDS* peritoneal dialysis solution, *pMSCs* MSCs in PDS, *PMC* peritoneal mesothelial cell, *EVs* extracellular vesicles, *UC-MSCs* umbilical cord-derived MSCs, *MMT* mesothelial–mesenchymal transition, *IDO* indoleamine 2,3-dioxygenase, *USCs* urine-derived SCs, *PGE2* prostaglandin E2, *HSCs* haematopoietic SCs, *AFSCs* amniotic fluid SCs

### Stem cells from different sources

There are many sources from which SCs can be collected, including bone marrow, placenta, the umbilical cord, amniotic fluid, adipose tissue, liver and muscle [51–53]. Numerous studies have demonstrated how SCs from various sources can alleviate PF, indicating potential applications in the treatment of PF.

MSCs have received the most research attention as a result of their general availability and ease of isolation, culture, expansion and preparation in high numbers for use in treatments. Bone marrow MSCs (BM-MSCs) and adipose-derived MSCs (AD-MSCs) are now the most widely studied and used SCs. Intraperitoneal infusion of BM-MSCs can inhibit inflammation and ameliorate PF by decreasing TGF-β1-induced MMT via paracrine effects [54]. TGF-β1 has been shown to be a robust profibrotic cytokine and the most prominent inducer of MMT in tissues and organs. When its effect is blocked by inhibitory peptides, the morphology and functionality of the peritoneum are protected. Moreover, Costalonga et al. [55] showed that AD-MSCs exhibited antifibrotic and anti-inflammatory immunomodulatory effects to inhibit PF in a uraemic rat PF model. Suppression of MMT by AD-MSCs in the early stages of tissue repair in an experimental PF model has also been demonstrated [56], with significant downregulation of the mRNA expression of MMT markers such as tumour necrosis factor α (TNF-α), IL-1β, monocyte chemotactic protein-1 (MCP-1), snail and α-SMA. A clinical study by Alatab et al. [57] showed that autologous AD-MSCs alleviated fibrotic conditions in PD patients with expected PF, verifying the feasibility and safety of AD-MSCs for use in PD patients. In addition, regarding the potential differences in treatments using the two most widely studied MSCs, in vivo rat experiments by Yang et al. [43] have revealed that AD-MSCs and BM-MSCs attenuate dialysis-induced PF and that AD-MSCs induce a more pronounced ameliorative effect than BM-MSCs on PM thickening while also upregulating epithelial cell markers in rat peritoneal tissues. These studies demonstrate that both AD-MSCs and BM-MSCs have significant and universal anti-PF effects, but it seems that AD-MSCs are more effective and have progressed faster in clinical application.

The role of umbilical cord-derived MSCs (UC-MSCs) in alleviating PF is also well known. However, even when the umbilical cord is the source, there is a degree of variability among MSCs from different parts of the cord, such as the umbilical cord membrane, subumbilical cord membrane, perivascular cord and Wharton's jelly; Wharton's jelly has the highest MSC levels and the MSCs with the greatest proliferative capacity. Experimental studies [58] have shown that direct intraperitoneal transplantation of human UC-MSCs from Wharton's jelly (WJ-MSCs) into rats inhibits the development of peritoneal thickening, fibrosis and inflammation caused by PD and methylglyoxal. Moreover, Dymowska et al. [59] discovered that WJ-MSCs can interact with macrophages in a reciprocal manner, driving M1-like (i.e. classically activated) proinflammatory macrophages towards the M2-like (i.e. alternatively activated) anti-inflammatory phenotype. In addition, compared to BM-MSCs, WJ-MSCs are significantly more responsive to macrophage chemotactic signals, demonstrating that the source of SCs can slightly but significantly alter SC behaviour. The safety and efficacy of the clinical use of UC-MSCs are being researched. A clinical study by Jiang et al. [60] revealed that UC-MSC treatment partially improved clinical indicators in patients undergoing continuous ambulatory PD (CAPD). Researchers have also discovered that UC-MSC treatment can attenuate inflammatory reactions and increase the level of EPO, which has antioxidant, antiapoptotic and anti-inflammatory properties [61]. The anti-PF effects of UC-MSCs have been fully verified in basic experiments, while clinical applications have demonstrated only that UC-MSCs have a certain anti-inflammatory effect in PD patients; their possible antifibrotic effect has not been directly confirmed. Thus, further research is needed.

Among SCs from the aforementioned sources, MSCs isolated from adipose tissue and bone marrow must be obtained by invasively, and MSCs isolation from the umbilical cord may raise ethical issues, thus limiting the clinical availability of these SCs. Recently, the presence of MSCs in PDS (called pMSCs) has been described [62]. pMSCs express essential markers and exhibit the biological characteristics of MSCs [63], suggesting that discarded PDS can provide a practical source of MSCs for large-scale clinical applications due to the large pool of PD patients. Moreover, pMSCs have also been shown to alleviate PF. In a rat model of biologically incompatible PDS induction, treatment with pMSCs prevented ultrafiltration loss and reduced PM injury and vascularity. In vitro incubation with pMSC-conditioned medium (CM) prevented death in cultured human PMCs (HPMCs) and decreased proinflammatory gene expression while increasing anti-inflammatory gene expression [64]. A study by Du et al. [36] published in 2021 showed that compared to UC-MSCs, pMSCs may more potently prevent PDS-induced PM injury; this effect is associated with higher resistance of pMSCs than UC-MSCs to uraemic toxins in culture and a greater reduction in PMC death by the secretome and soluble factors from pMSCs than by those from UC-MSCs in response to PDS exposure. The pMSCs represent a relatively novel type of SCs that are closely related to the peritoneum, and their relatively convenient accessibility and considerable therapeutic effects have made them promising options for anti-PF approaches. Thus, pMSCs are worthy of more in-depth studies and attempts at clinical application.

Some of the more well-known SCs studied in recent years are urine-derived SCs (USCs). USCs are populations of urine-derived cells with the capacity for multidirectional differentiation whose biological characteristics are comparable to those of MSCs and embryonic SCs. USCs have several advantages over the MSCs mentioned previously, including the following: (1) they can be obtained regardless of a person's age, sex or health status (except for those with urinary tract infections and anuria); (2) they can be collected using simple, safe, low-cost and noninvasive procedures; (3) pure SCs are easier to isolate without the need for an enzymatic digestion process; and (4) they show telomerase activity, enabling them to produce more cells than other SC types [65]. Additionally, as has been repeatedly described for MSCs, USCs also exhibit immunomodulatory properties [66]. Kang et al. [67] compared USCs to AD-MSCs and discovered that USCs had greater proliferation capacity, higher expression of SC markers and greater immune cell suppression efficiency. Furthermore, it has been demonstrated that USCs exert tissue-protective effects via anti-inflammatory, anti-oxidative stress and antifibrotic mechanisms. USCs significantly reduce the expression of the fibrogenic factor TGF-β1 and elevate the expression of superoxide dismutase 1 (SOD-1), an important mediator that inhibits free radical-induced tissue damage, in mice with chronic kidney disease [68]. USCs suppress Th1 and Th17 immune responses in rodent models of inflammatory bowel disease, which further demonstrates their capacity to reduce inflammation and prevent fibrosis [49]. Studies in recent years [48, 69, 70] have also shown the involvement of USCs and their paracrine compounds in reducing bladder, cardiac, penile and glomerular fibrosis, but these studies have not examined the roles of USCs in other types of tissue fibrosis. The immunomodulatory and antifibrotic properties of USCs may contribute to PF, but additional research is needed to confirm this hypothesis.

Furthermore, there are also other SCs that contribute to reducing tissue fibrosis. Shen et al. [71] identified a new source of autologous human haematopoietic SCs (HSCs) in PDS from patients with end-stage renal disease (ESRD) and found that, in contrast to cord blood-derived HSCs, PD-derived HSCs were able to repopulate the peritoneal cavity with myeloid cells and B lymphocytes. This may benefit the immune response to infections and play a role in combating frequent peritonitis. HSCs are bone marrow-derived cells that can differentiate into MCs for peritoneal repair and remodelling [34]. The effects of HSCs alone on PF have not been reported, but their function in alleviating the progression of liver fibrosis has been studied [72], and HSCs are known to inhibit fibrosis and apoptosis by secreting bioactive factors that suppress the local immune response. We therefore hypothesize that HSCs may affect PF, which needs to be confirmed by further studies.

Amniotic fluid is another source of SCs [50], and paracrine factors released by human amniotic fluid SCs (hAFSCs) induce M1-M2 macrophage polarization in a cell contact-independent manner, showing anti-inflammatory effects.

These animal and clinical studies suggest that SCs may alleviate PF and be effective treatment options. The efficacy of SCs in combating fibrosis varies slightly among SCs from different sources, so thorough research on more practical and convenient SC therapies is warranted.

### Pretreatments enhance the therapeutic effects of stem cells

When SCs are cultured in vitro, the culture medium and additives (such as glucose, growth factors, lipids and vitamins), as well as the culture conditions (such as the oxygen concentration in the incubator, the presence of cell-dissociating agents and the density of inoculated cells), typically affect the proliferative capacity and activity of SCs [73]. In addition, the hostile microenvironment created by in vivo transplantation severely reduces SC regeneration. As a result, primary SCs or unprepared SCs frequently fail to produce the desired therapeutic effects because of poor homing capacity, low posttransplant survival and cellular senescence or decreased viability during in vitro culture. Pretreatment with chemicals, cytokines, hypoxia and inflammatory factors, on the other hand, has garnered widespread attention as an enhancement strategy. Pretreatment stimulates SC activation by incorporating features of physiological and pathological microenvironments not only to protect SCs from damage but also to improve SC homing capacity, survival and paracrine effects in vitro and in vivo [74, 75], thereby improving the ability to alleviate PF.

A study by Nagasaki et al. [44] published in 2021 showed that, compared with MSCs cultured in medium containing 10% foetal bovine serum (FBS), MSCs pretreated with serum-free culture conditions exhibited enhanced antifibrotic ability. This enhancement was mediated by suppression of inflammatory cell infiltration through upregulation of the expression of TNF-α-induced protein 6 (TSG-6). TSG-6 secreted by MSCs has been reported to play an important role in suppressing the infiltration of inflammatory cells induced by tissue injury at an early phase [76, 77]. In addition, the pretreated MSCs more effectively induced the conversion of M1 macrophages to M2 macrophages.

Philipp et al. [40] found that murine MSCs that were preconditioned with IL-1β and IFN-γ could release large quantities of NO, IL-6 and prostaglandin E2 (PGE2), while IL-6 could significantly enhance the ability of these cells to regulate macrophage characteristics and induce immunosuppression. Other studies [78] have supported this combination technique; for example, pretreatment of human umbilical cord blood-derived MSCs (hUCB-MSCs) with IL-1β and IFN-γ has been found to increase PGE2 secretion and indoleamine 2,3-dioxygenase (IDO) activity and increase the ability of hUCB-MSCs to modulate the immune system.

Han et al. [63] characterized pMSCs after expansion in human protein culture medium rather than conventional FBS-containing culture medium, which may pose several safety concerns, and showed that isolated pMSCs could expand into spheroids. Studies have revealed that SC spheroids significantly increase the therapeutic potential of SCs, enhancing anti-inflammatory effects and promoting tissue regeneration and repair [79]. This raises the possibility of mass producing pMSC spheroids for use in treating PD and other clinical applications.

Additionally, one study has reported [45] that MSCs pretreated with *Leishmania major* soluble antigens can induce anti-inflammatory properties in mouse peritoneal macrophages.

Various pretreatment methods have been shown to improve the capacity and therapeutic efficacy of SCs, so cell-based pretreatment is a promising topic for future research.

### Genetic modification enhances the therapeutic effects of stem cells

To increase SC differentiation, immunomodulation, homing and other repair-related capacities, a variety of genes and microRNAs with clearly defined biological activities can be inserted into SCs using viral or nonviral vectors. The combination of SC therapies and biotechnology is a promising area in tissue damage and wound healing.

SIRT1 is a nicotinamide adenosine dinucleotide (NAD)-dependent deacetylase that is involved in regulating a variety of physiological functions and has been shown to have a therapeutic role in PF [80]. Guo et al. [47] overexpressed SIRT1 in human umbilical cord-derived MSCs (hUC-MSCs) and demonstrated that SIRT1-modified hUC-MSCs exhibit improved paracrine and posttranscriptional modification abilities that attenuate peritoneal inflammation and fibrosis through the TGF-β/Smad3 pathway.

In addition, genetically modifying SCs can enhance their immunomodulatory effects, thereby increasing their therapeutic effects. For instance, MSCs overexpressing IL-35 have an increased immunosuppressive capacity, as IL-35-MSCs have been shown to induce CD4 + T cells to produce the anti-inflammatory factor IL-10 [81, 82]. The therapeutic effects of MSCs on organ injury and inflammation are also enhanced by overexpression of TGF-β1 due to a decrease in macrophage infiltration and induction of macrophage phenotypic transformation, and overt transfer of macrophages treated with TGF-β1-overexpressing MSCs reduces organ injury [83]. Therefore, modifying certain genes is a potential new approach to treating PF.

## Mechanisms of stem cell-based therapy

The major mechanisms of SC therapy for PF involve paracrine activity to accelerate healing or differentiation into functional repair cells such as MCs that replace the injured tissue. According to previous studies, paracrine action may be the more important mode of action; in this mechanism, SCs either directly act on MCs by secreting cytokines, extracellular vesicles (EVs), etc., or indirectly act through immunomodulatory mechanisms to alleviate PF. The potential mechanisms of SC-based therapy in PF are shown in Fig. 2. We also list the possible mechanisms of the major therapeutic strategies for SCs and their recent relevant studies in Table 1.

### Settlement and differentiation

SCs have the potential for plasticity and multidirectional differentiation. SCs migrate to the site of tissue damage and differentiate to replace damaged cells in response to certain chemokines, thus acting as tissue regenerators and repairers. Fan et al. [58] significantly alleviated PF in a methylene glycol-induced rat model by intraperitoneally injecting the rats with WJ-MSCs. Surviving MSCs were found in the peritoneum in the rats 3 weeks after injection, indicating that the SCs could migrate into injured peritoneal tissue to exert their reparative effects. Additionally, after colonizing injured tissues, SCs can develop into specific functional cells and begin exerting their effects. Chen et al. [84] found that transplanted bone marrow-derived SCs not only attached to the peritoneum but also changed morphologically into PMCs. Bone marrow-derived SCs include MSCs, haematopoietic SCs, epithelial progenitor cells and others, and transplantation of these cells can be used to treat PF. Sekiguchi et al. [34] further demonstrated that c-Kit-positive bone marrow-derived SCs were detectable in the peritoneum in PF model mice with chlorhexidine gluconate (CG)-induced injury and were able to differentiate into MCs that participated in peritoneal repair and remodelling. It is therefore clear that the direct migration and multidirectional differentiation potential of SCs play a role in the repair of peritoneal injury. However, it has been noted that after intravenous SC injection, cells primarily accumulate in the endothelium of lung vessels [85]. Similarly, SCs that are intraperitoneally injected into the injured peritoneal region do not significantly colonize the area of injury but are found mainly in the liver and spleen, suggesting that settlement and differentiation may not be the main mechanisms of action of SCs.

### Effects of stem cell-derived cytokines

As research progresses, it is becoming more evident that the paracrine effects of SCs may be more important than their multidirectional differentiation potential [86]. By secreting various cytokines, SCs act on MCs and inhibit MMT to ameliorate PF. Several studies [40, 41, 87–89] have demonstrated that SCs can produce a wide range of cytokines, such as hepatocyte growth factor (HGF), IL-6, IL-4, IL-10, TSG-6 and IDO. Table 2 lists the major cytokines secreted by SCs that mediate their functions and the corresponding receptors and receptor cells. The cytokines that act on immune cells will be emphasized later in the section on immunoregulatory mechanisms.

**Table 2:** Major cytokines produced by stem cells and their receptors

Cytokine	Secretory cell	Cytokine receptor	Receptor cell
TSG-6	MSCs [87, 90], AFSCs [50, 89]	CD44 [90]	Macrophages
HGF	AD-MSCs [35]	c-Met [91]	Mesothelial cells
IL-6	AD-MSCs [43], BM-MSCs [40]	IL-6R [40]	Macrophages
PGE2	MSCs [92]	EP2, EP4 [93]	Macrophages
	USCs [49]	–	CD4 + T cells
IDO	BM-MSCs [41]	–	–
TGF-β	MSCs [94]	TGF-βR	Macrophages

A brief list of the major cytokines secreted by stem cells in attenuating peritoneal fibrosis and the corresponding receptors and receptor cells

*SCs* stem cells, *MSCs* mesenchymal stem cells, *TSG-6* tumour necrosis factor-α-induced protein 6, *AD-MSCs* adipose-derived MSCs, *AFSCs* amniotic fluid SCs, *HGF* hepatocyte growth factor, *c-Met* cellular mesenchymal–epithelial transition factor, *BM-MSCs* bone marrow MSCs, *PGE2* prostaglandin E2, *EP2* prostaglandin E2 receptor 2, *EP4* prostaglandin E2 receptor 4, *USCs* urine-derived SCs, *IDO* indoleamine 2,3-dioxygenase

Wang et al. [87] found that MSCs can attenuate peritoneal injury by repairing MCs and reducing inflammation and fibrosis via secretion of TSG-6.

HGF is an antifibrotic cytokine that exerts therapeutic potential by interfering with TGF-β1 signalling and preventing tissue fibrosis [95]. Experimental studies [35, 96] have confirmed that SCs release HGF, which reduces peritoneal adhesion and fibrosis by promoting the migration and proliferation of MCs; the mechanism may involve an antiapoptotic effect of HGF that reduces peritoneal tissue damage [91].

Furthermore, SCs can exert anti-inflammatory effects by increasing the levels of anti-inflammatory cytokines (IL-10, etc.) and decreasing those of proinflammatory cytokines (IL-1a, IL-6, IL-17, TNF-α, IFN-γ, etc.) [97], thereby reducing peritoneal damage.

### Effects of stem cell-derived extracellular vesicles

SCs can also play paracrine roles by secreting EVs. Based on the size of the vesicles and the method of cellular release, EVs can be broadly classified into three subtypes: exosomes (30–130 nm), microvesicles (100–1000 nm) and apoptotic vesicles (50–4000 nm) [98]. However, there is a lack of a unique method to distinguish among the three subtypes of EVs due to the overlapping size, density and membrane composition of the EVs [99]. SC-derived EVs are considered a cell-free product and can be obtained from the conditioned medium of SCs [100]. Several studies have shown [101, 102] that EVs transmit proteins, lipids and nucleic acids between various cell types, participate in critical biological processes and mediate cell-to-cell communication. Additionally, in some circumstances, SC-EVs are therapeutically equivalent to directly infused SCs and may be more advantageous than SCs alone in practical applications due to their lower immunogenicity [103], ease of preservation and transfer and ease of production [104]. EVs are the main vehicles associated with SC activity and can interact with MCs to mitigate peritoneal damage and excessive repair.

Some studies have shown that ADSC-EVs can be incorporated in vitro and induce rat PMC proliferation and migration, which is mediated by stimulation of the MAPK–ERK1/2 and PI3K–AKT axes. ADSC-EVs promote healing of the injured peritoneum, suggesting that they are promising therapeutic tools for peritoneal adhesions [38].

Previous studies [105] have found that EVs contain packaged microRNAs (miRNAs) that are involved in the regulation of cells. Further research has revealed the crucial functions of SC-secreted exosomes containing miRNAs and long noncoding RNAs (lncRNAs) in the treatment of fibrosis. For instance, miR-34c-5p secreted from BM-MSC-derived exosomes (BM-MSC-Exos) ameliorates renal interstitial fibrosis by inhibiting multicellular activation via core fucosylation [106]. MSC-derived exosomal miR-466f-3p reverses epithelial–mesenchymal transition in lung fibrosis through inhibition of AKT/GSK3β [107]. MiR-196b-5p in MSC-derived exosomes significantly inhibits collagen type I alpha 2 expression in fibroblasts [108].

Similarly, recent research has found that exosomes and multiple cargoes they deliver play effective therapeutic roles in PF. Yu et al. [37] published a new study in April 2023 showing that BM-MSC-Exos carrying a series of miRNAs of different functions affect the cell cycle process, cell differentiation and inflammatory response regulation, which can effectively prevent PF and peritoneal injury. However, the authors also suggested that the specific mechanisms of action of the different miRNAs are still unclear and require more in-depth research. Meanwhile, a recent study by Huang et al. [39] has revealed that the exosomal lncRNA GAS5 derived from UC-MSCs can reduce MMT through the Wnt/β-catenin signalling pathway. Another emerging study [109] focusing on lncRNA has also suggested a role for exosomal lncRNA in alleviating PF, showing that the lncRNA CDHR derived from UC-MSC-Exos attenuates MMT through the AKT/FOXO pathway. All of these findings highlight the functions and molecular mechanisms of EVs in PF, especially exosomal miRNAs and lncRNAs, which exhibit promising research prospects.

### Immunoregulation of stem cells

SCs have broad immunosuppressive potential and can modulate the activity of innate and adaptive immune cells via intercellular contact or paracrine effects. Furthermore, there is mounting evidence that paracrine factors from SCs can modulate the immune system to attenuate PF by interacting with a wide range of immune cells [49, 110], particularly macrophages.

Macrophages are one of the key cell types linking the innate and adaptive immune systems. M1 macrophages exert proinflammatory effects through the production of proinflammatory cytokines and protein hydrolases, while M2 macrophages have anti-inflammatory effects and contribute to inflammation regression and tissue remodelling [111]. According to research [112], M1 macrophages are important mediators of PF. SCs have been shown to promote the polarization of M1 macrophages into M2 macrophages, which then exert immunosuppressive and anti-inflammatory effects to mitigate the progression of PF [113].

A study by Yang et al. [43] has shown that MSCs situated in an inflammatory environment with TGF-β1 can secrete IL-6 to polarize macrophages into the M2 phenotype in dialysis-induced PF. Similarly, Philipp et al. [40] demonstrated that MSC-dependent polarization towards the M2 phenotype in a proinflammatory microenvironment and towards the regulatory M2b phenotype in the presence of anti-inflammatory IL-4 strongly depends on functional IL-6 signalling. To determine the role of IL-6 in MSC-macrophage interactions, Dymowska et al. [59] blocked IL-6 signalling and found that IL-6 does not drive the MSC-mediated switch in macrophages into the M2 phenotype. However, different experiments have used different sources of tissue-specific SCs, which may have resulted in the variety of outcomes. Furthermore, the effects of MSCs on macrophages are influenced by more than one factor. It is unclear how different mediators interact with one another; they may have complementary effects, but some data suggest that there are also overlapping effects [114]. Therefore, excluding one factor may not significantly affect the functional result, leading to different experimental results.

Bioactive substances such as PGE2 in the induced SC secretome, which affect the metabolic frameworks of differently polarized macrophages and direct M1–M2 polarization shifts, play crucial mechanistic roles in modulating the metabolic status and plasticity of macrophages [92, 93, 115]. PGE2 levels are markedly upregulated in MSC cocultures and MSC-EVs, causing decreases in IL-23 and IL-22 production and thereby enhancing the anti-inflammatory phenotype of mature human regulatory macrophages (Mregs) [116].

François et al. [41] demonstrated that human BM-MSCs play a central role in inducible IDO activity and its bystander effect on M2 macrophage differentiation. Liu et al. [94] found that TGF-β, a cytokine secreted by MSCs, can skew lipopolysaccharide (LPS)-stimulated macrophage polarization towards the M2 phenotype and suppress inflammatory reactions. TSG-6 secreted by SCs has also been shown to act on macrophages, promote M2 macrophage polarization and reduce inflammatory responses [50, 117–119]. Additionally, it has been demonstrated that MSCs interact with CD44 receptors on resident macrophages in the peritoneum via TSG-6 to reduce enzymatic glycan/TLR2-mediated NF-κB nuclear translocation, thereby inhibiting the inflammatory cascade initiated by resident macrophages and reducing peritoneal inflammation [90]. All of these SC-secreted cytokines have been shown to act on macrophages to exert immunomodulatory effects in order to reduce peritoneal inflammation and thus prevent the development of PF. However, the specific mechanisms of action of these cytokines are still unclear, and few studies have focused on this topic, which is worth further investigation.

In addition, MSC-derived exosomes have been shown to promote M2 macrophage polarization and exert immunosuppressive effects [42]. Melatonin-pretreated MSC-derived exosomes (MT-Exos) exert enhanced immunomodulatory effects on macrophages by increasing the ratio of M2-to-M1 polarization, possibly by activating the PTEN/AKT signalling pathway, upregulating PTEN expression and inhibiting AKT phosphorylation, which suppresses inflammatory responses [46].

## Conclusions and future perspectives

PF is a pathophysiological process caused by a variety of pathogenic factors and abnormal proliferation, with the most prominent feature being MMT and accumulation of activated (myo-)fibroblasts. In recent years, the therapeutic potential of SCs and their secretomes for tissue damage repair, immunomodulation and inflammation inhibition has been actively assessed in many different pathologies [120, 121], including peritoneal inflammation and fibrosis [122].

Currently, most of the basic experiments and clinical studies have shown that many kinds of SCs from different sources can effectively alleviate PF. Whether through cellular differentiation or by exerting immune regulation and cellular repair effects, SCs are suggested as a new strategy for the clinical treatment of PF. And pretreatment or genetic modification of SCs has been demonstrated to further enhance their therapeutic effects. Notably, traditional Chinese medicine (TCM) offers a unique approach to the treatment of complex chronic diseases. It has been shown that TCMs such as astragalus, rhubarb, safflower and curcumin exert significant therapeutic effects on peritoneal injury as well as regulating inflammation and inhibiting abnormal ECM accumulation [123–126]. And SCs pretreated with TCMs have been proven to play a therapeutic role in many diseases [127, 128]. However, there are no relevant studies on the combination of TCMs and SCs to treat PF; thus, SC preconditioning with TCMs may be a future research direction.

Meanwhile, although a significant amount of basic research has confirmed the effective role of SCs in alleviating PF, there are still controversies and problems with their widespread clinical application. First, the select of appropriate SC types and sources is an urgent challenge. SCs contain many types and can be derived from a variety of tissues, such as bone marrow, adipose tissue, umbilical cords, PDS, urine and amniotic fluid. The biological properties and therapeutic effects of different kinds of SCs from different tissues vary, while each form of stem cell encounters some challenges when used in clinical therapy. BM-MSCs, which are the most widely known among MSCs, are more traumatic to the body and more difficult to identify and extract than other types, and AD-MSCs are easy to obtain but require invasive manipulation. In addition, the application of UC-MSCs involves complex medical ethical issues. The pMSCs have certain application prospects, but relatively few studies have been conducted thus far, and the feasibility of their clinical application is not yet known. The practical use of USCs and hAFSCs is still very limited, particularly in the field of PF, and the related mechanisms and adverse effects are not yet apparent. Therefore, the selection of effective and abundantly sourced SCs is the primary problem faced in clinical treatment. Meanwhile, there is a lack of uniform and standardized methods for the isolation, culture and identification of SCs from various sources, all of which need to be further explored. Currently, research on the solution is gradually expanding, and we believe that the problem will be solved in the future.

On the other hand, there is no consensus on the transplantation protocol for SCs. Up to now, clinical studies related to the transplantation of SCs into human beings in PF are limited, and no uniform standards have been established on how to choose the transplantation route, the transplantation dose, the number of transplants and the time of post-transplantation survival, so optimizing the standardized protocols for SC transplantation is particularly important for clinical treatment. For the transplantation route, there are mainly intraperitoneal injection and intravenous injection. Compared with intravenous injection, intraperitoneal injection can better reduce the inflammatory response, repair peritoneal injury and improve peritoneal function [129], but this is only the experimental results in the rat model, while the comparison of the merits and demerits of the application in humans is not yet known. At the same time, there are no studies on the survival time of SCs after transplantation into humans. Therefore, how to draw up the SC transplantation protocol is also a major difficulty in clinical application. Nevertheless, in addition to the infusion of SCs themselves, derivatives such as EVs have the advantages of low immunogenicity and low tumorigenicity, and a large number of research is being conducted in this area, which is likely to become an important clinical direction.

We firmly believe that SC therapy has a great deal of promise for alleviating PF and that SCs derived from various sources that are pretreated or prepared with various genetic alterations could be valuable research topics in the coming years. However, since there are currently no standardized approach for treatments using SCs and no consensus on transplantation protocols for SCs, it will be challenging to implement SC-based PF treatments in actual clinical settings soon. Further study on SC therapies is definitely needed before these cells can be routinely used in clinical practice.

## Data Availability

Not applicable.

## Abbreviations

PF: Peritoneal fibrosis
MMT: Mesothelial–mesenchymal transition
SC: Stem cell
PD: Peritoneal dialysis
PMC: Peritoneal mesothelial cell
ECM: Extracellular matrix
PM: Peritoneal membrane
PDS: Peritoneal dialysis solution
MSCs: Mesenchymal stem cells
MCs: Mesothelial cells
ZO-1: Zonula occludens-1
FSP1: Fibroblast-specific protein-1
α-SMA: α-Smooth muscle actin
PRRs: Pattern recognition receptors
PAMPs: Pathogen-associated molecular patterns
EGFR: Epidermal growth factor receptor
GDPs: Glucose degradation products
AGEs: Advanced glycosylation end products
ROS: Reactive oxygen species
DAMPs: Damage-associated molecular patterns
TLRs: Toll-like receptors
NF-κB: Nuclear factor-κB
TGF-β1: Transforming growth factor-β1
VEGF: Vascular endothelial growth factor
HGPDS: High-glucose PDS
BM-MSCs: Bone marrow MSCs
AD-MSCs: Adipose-derived MSCs
TNF-α: Tumour necrosis factor α
MCP-1: Monocyte chemotactic protein-1
UC-MSCs: Umbilical cord-derived MSCs
WJ-MSCs: UC-MSCs from Wharton's jelly
CAPD: Continuous ambulatory PD
pMSCs: MSCs in PDS
CM: Conditioned medium
HPMCs: Human PMCs
USCs: Urine-derived SCs
SOD-1: Superoxide dismutase 1
HSCs: Haematopoietic SCs
ESRD: End-stage renal disease
hAFSCs: Human amniotic fluid SCs
FBS: Foetal bovine serum
TSG-6: Tumour necrosis factor-α-induced protein 6
PGE2: Prostaglandin E2
EP2: Prostaglandin E2 receptor 2
EP3: Prostaglandin E2 receptor 4
hUCB-MSCs: Human umbilical cord blood-derived MSCs
IDO: Indoleamine 2,3-dioxygenase
NAD: Nicotinamide adenosine dinucleotide
hUC-MSCs: Human umbilical cord-derived MSCs
EVs: Extracellular vesicles
CG: Chlorhexidine gluconate
HGF: Hepatocyte growth factor
LPS: Lipopolysaccharide
MT-Exos: Melatonin-pretreated MSC-derived exosomes
TCM: Traditional Chinese medicine

## References

[1] Li PK-T, Chow KM, Van de Luijtgaarden MWM, Johnson DW, Jager KJ, Mehrotra R (2017). Changes in the worldwide epidemiology of peritoneal dialysis. Nat Rev Nephrol.

[2] Cho Y, Johnson DW (2014). Peritoneal dialysis-related peritonitis: towards improving evidence, practices, and outcomes. Am J Kidney Dis.

[3] Devuyst O, Margetts PJ, Topley N (2010). The pathophysiology of the peritoneal membrane. J Am Soc Nephrol JASN.

[4] Silva FMO, Costalonga EC, Silva C, Carreira ACO, Gomes SA, Sogayar MC (2019). Tamoxifen and bone morphogenic protein-7 modulate fibrosis and inflammation in the peritoneal fibrosis model developed in uremic rats. Mol Med.

[5] Wang Y, Shi Y, Tao M, Zhuang S, Liu N (2021). Peritoneal fibrosis and epigenetic modulation. Perit Dial Int.

[6] Zakrzewski W, Dobrzyński M, Szymonowicz M, Rybak Z (2019). Stem cells: past, present, and future. Stem Cell Res Ther.

[7] Mutsaers SE, Birnie K, Lansley S, Herrick SE, Lim C-B, Prêle CM (2015). Mesothelial cells in tissue repair and fibrosis. Front Pharmacol.

[8] Wynn TA, Ramalingam TR (2012). Mechanisms of fibrosis: therapeutic translation for fibrotic disease. Nat Med.

[9] Krenning G, Zeisberg EM, Kalluri R (2010). The origin of fibroblasts and mechanism of cardiac fibrosis. J Cell Physiol.

[10] Loureiro J, Aguilera A, Selgas R, Sandoval P, Albar-Vizcaíno P, Pérez-Lozano ML (2011). Blocking TGF-β1 protects the peritoneal membrane from dialysate-induced damage. J Am Soc Nephrol.

[11] Liu Y, Dong Z, Liu H, Zhu J, Liu F, Chen G (2015). Transition of mesothelial cell to fibroblast in peritoneal dialysis: EMT, stem cell or bystander?. Perit Dial Int.

[12] Debnath P, Huirem RS, Dutta P, Palchaudhuri S (2022). Epithelial–mesenchymal transition and its transcription factors. Biosci Rep.

[13] Aufricht C, Beelen R, Eberl M, Fischbach M, Fraser D, Jörres A (2017). Biomarker research to improve clinical outcomes of peritoneal dialysis: consensus of the European Training and Research in Peritoneal Dialysis (EuTRiPD) network. Kidney Int.

[14] Ragland SA, Kagan JC (2021). Cytosolic detection of phagosomal bacteria—mechanisms underlying PAMP exodus from the phagosome into the cytosol. Mol Microbiol.

[15] Goodlad C, George S, Sandoval S, Mepham S, Parekh G, Eberl M (2020). Measurement of innate immune response biomarkers in peritoneal dialysis effluent using a rapid diagnostic point-of-care device as a diagnostic indicator of peritonitis. Kidney Int.

[16] Jagirdar RM, Bozikas A, Zarogiannis SG, Bartosova M, Schmitt CP, Liakopoulos V (2019). Encapsulating peritoneal sclerosis: pathophysiology and current treatment options. Int J Mol Sci.

[17] Tomita T, Arai S, Kitada K, Mizuno M, Suzuki Y, Sakata F (2017). Apoptosis inhibitor of macrophage ameliorates fungus-induced peritoneal injury model in mice. Sci Rep.

[18] Zindel J, Mittner J, Bayer J, April-Monn SL, Kohler A, Nusse Y (2021). Intraperitoneal microbial contamination drives post-surgical peritoneal adhesions by mesothelial EGFR-signaling. Nat Commun.

[19] Kang D-H (2020). Loosening of the mesothelial barrier as an early therapeutic target to preserve peritoneal function in peritoneal dialysis. Kidney Res Clin Pract.

[20] Mortier S, Faict D, Schalkwijk CG, Lameire NH, De Vriese AS (2004). Long-term exposure to new peritoneal dialysis solutions: effects on the peritoneal membrane. Kidney Int.

[21] Si M, Wang Q, Li Y, Lin H, Luo D, Zhao W (2019). Inhibition of hyperglycolysis in mesothelial cells prevents peritoneal fibrosis. Sci Transl Med..

[22] Helmke A, Hüsing AM, Gaedcke S, Brauns N, Balzer MS, Reinhardt M (2021). Peritoneal dialysate-range hypertonic glucose promotes T-cell IL-17 production that induces mesothelial inflammation. Eur J Immunol.

[23] Roh JS, Sohn DH (2018). Damage-associated molecular patterns in inflammatory diseases. Immune Netw.

[24] Raby A-C, González-Mateo GT, Williams A, Topley N, Fraser D, López-Cabrera M (2018). Targeting Toll-like receptors with soluble Toll-like receptor 2 prevents peritoneal dialysis solution-induced fibrosis. Kidney Int.

[25] Balzer MS (2020). Molecular pathways in peritoneal fibrosis. Cell Signal.

[26] Yung S, Chan TM (2012). Pathophysiological changes to the peritoneal membrane during PD-related peritonitis: the role of mesothelial cells. Mediators Inflamm.

[27] Wu J, Xing C, Zhang L, Mao H, Chen X, Liang M (2018). Autophagy promotes fibrosis and apoptosis in the peritoneum during long-term peritoneal dialysis. J Cell Mol Med.

[28] Zhang Z, Jiang N, Ni Z (2017). Strategies for preventing peritoneal fibrosis in peritoneal dialysis patients: new insights based on peritoneal inflammation and angiogenesis. Front Med.

[29] Terri M, Trionfetti F, Montaldo C, Cordani M, Tripodi M, Lopez-Cabrera M (2021). Mechanisms of peritoneal fibrosis: focus on immune cells-peritoneal stroma interactions. Front Immunol.

[30] Santos A, Lagares D (2018). Matrix stiffness: the conductor of organ fibrosis. Curr Rheumatol Rep.

[31] Sandoval P, Jiménez-Heffernan JA, Guerra-Azcona G, Pérez-Lozano ML, Rynne-Vidal Á, Albar-Vizcaíno P (2016). Mesothelial-to-mesenchymal transition in the pathogenesis of post-surgical peritoneal adhesions. J Pathol.

[32] Strippoli R, Sandoval P, Moreno-Vicente R, Rossi L, Battistelli C, Terri M (2020). Caveolin1 and YAP drive mechanically induced mesothelial to mesenchymal transition and fibrosis. Cell Death Dis.

[33] Bacakova L, Zarubova J, Travnickova M, Musilkova J, Pajorova J, Slepicka P (2018). Stem cells: their source, potency and use in regenerative therapies with focus on adipose-derived stem cells—a review. Biotechnol Adv.

[34] Sekiguchi Y, Hamada C, Ro Y, Nakamoto H, Inaba M, Shimaoka T (2012). Differentiation of bone marrow-derived cells into regenerated mesothelial cells in peritoneal remodeling using a peritoneal fibrosis mouse model. J Artif Organs.

[35] Kim H, Mizuno M, Furuhashi K, Katsuno T, Ozaki T, Yasuda K (2014). Rat adipose tissue-derived stem cells attenuate peritoneal injuries in rat zymosan-induced peritonitis accompanied by complement activation. Cytotherapy.

[36] Du Y, Zong M, Guan Q, Huang Z, Zhou L, Cai J (2021). Comparison of mesenchymal stromal cells from peritoneal dialysis effluent with those from umbilical cords: characteristics and therapeutic effects on chronic peritoneal dialysis in uremic rats. Stem Cell Res Ther.

[37] Yu F, Yang J, Chen J, Wang X, Cai Q, He Y (2023). Bone marrow mesenchymal stem cell-derived exosomes alleviate peritoneal dialysis-associated peritoneal injury. Stem Cells Dev.

[38] Shi M, Liu H, Zhang T, Zhang M, Tang X, Zhang Z (2022). Extracellular vesicles derived from adipose mesenchymal stem cells promote peritoneal healing by activating MAPK-ERK1/2 and PI3K-Akt to alleviate postoperative abdominal adhesion. Stem Cells Int.

[39] Huang Y, Ma J, Fan Y, Yang L (2023). Mechanisms of human umbilical cord mesenchymal stem cells-derived exosomal lncRNA GAS5 in alleviating EMT of HPMCs via Wnt/β-catenin signaling pathway. Aging.

[40] Philipp D, Suhr L, Wahlers T, Choi Y-H, Paunel-Görgülü A (2018). Preconditioning of bone marrow-derived mesenchymal stem cells highly strengthens their potential to promote IL-6-dependent M2b polarization. Stem Cell Res Ther.

[41] François M, Romieu-Mourez R, Li M, Galipeau J (2012). Human MSC suppression correlates with cytokine induction of indoleamine 2,3-dioxygenase and bystander M2 macrophage differentiation. Mol Ther.

[42] He X, Dong Z, Cao Y, Wang H, Liu S, Liao L (2019). MSC-derived exosome promotes M2 polarization and enhances cutaneous wound healing. Stem Cells Int.

[43] Chang P-Y, Chen J-Y, Wu B-S, Yang A-H, Lee OKS, Yang C-Y (2021). Adipose-derived mesenchymal stem cells attenuate dialysis-induced peritoneal fibrosis by modulating macrophage polarization via interleukin-6. Stem Cell Res Ther.

[44] Nagasaki K, Nakashima A, Tamura R, Ishiuchi N, Honda K, Ueno T (2021). Mesenchymal stem cells cultured in serum-free medium ameliorate experimental peritoneal fibrosis. Stem Cell Res Ther.

[45] Khosrowpour Z, Hashemi SM, Mohammadi-Yeganeh S, Soudi S (2017). Pretreatment of mesenchymal stem cells with Leishmania major soluble antigens induce anti-inflammatory properties in mouse peritoneal macrophages. J Cell Biochem.

[46] Liu W, Yu M, Xie D, Wang L, Ye C, Zhu Q (2020). Melatonin-stimulated MSC-derived exosomes improve diabetic wound healing through regulating macrophage M1 and M2 polarization by targeting the PTEN/AKT pathway. Stem Cell Res Ther.

[47] Guo Y, Wang L, Gou R, Wang Y, Shi X, Pang X (2020). SIRT1-modified human umbilical cord mesenchymal stem cells ameliorate experimental peritoneal fibrosis by inhibiting the TGF-β/Smad3 pathway. Stem Cell Res Ther.

[48] Wu J, Wang X, Fu G, Feng Y, Wang Y, Zhang G (2023). Exosomes from human urine-derived stem cells carry NRF1 to alleviate bladder fibrosis via regulating miR-301b-3p/TGFβR1 pathway. Mol Cell Biochem.

[49] Zhou C, Wu X-R, Liu H-S, Liu X-H, Liu G-H, Zheng X-B (2020). Immunomodulatory effect of urine-derived stem cells on inflammatory bowel diseases via downregulating Th1/Th17 immune responses in a PGE2-dependent manner. J Crohns Colitis.

[50] Sato Y, Ochiai D, Abe Y, Masuda H, Fukutake M, Ikenoue S (2020). Prophylactic therapy with human amniotic fluid stem cells improved survival in a rat model of lipopolysaccharide-induced neonatal sepsis through immunomodulation via aggregates with peritoneal macrophages. Stem Cell Res Ther.

[51] Macrin D, Joseph JP, Pillai AA, Devi A (2017). Eminent sources of adult mesenchymal stem cells and their therapeutic imminence. Stem Cell Rev Rep.

[52] Chen J, Chen L, Zern MA, Theise ND, Diehl AM, Liu P (2017). The diversity and plasticity of adult hepatic progenitor cells and their niche. Liver Int.

[53] Zhang S, Fang J, Liu Z, Hou P, Cao L, Zhang Y (2021). Inflammatory cytokines-stimulated human muscle stem cells ameliorate ulcerative colitis via the IDO-TSG6 axis. Stem Cell Res Ther.

[54] Ueno T, Nakashima A, Doi S, Kawamoto T, Honda K, Yokoyama Y (2013). Mesenchymal stem cells ameliorate experimental peritoneal fibrosis by suppressing inflammation and inhibiting TGF-β1 signaling. Kidney Int.

[55] Costalonga EC, Fanelli C, Garnica MR, Noronha IL (2020). Adipose-derived mesenchymal stem cells modulate fibrosis and inflammation in the peritoneal fibrosis model developed in uremic rats. Stem Cells Int.

[56] Wakabayashi K, Hamada C, Kanda R, Nakano T, Io H, Horikoshi S (2014). Adipose-derived mesenchymal stem cells transplantation facilitate experimental peritoneal fibrosis repair by suppressing epithelial–mesenchymal transition. J Nephrol.

[57] Alatab S, Shekarchian S, Najafi I, Moghadasali R, Ahmadbeigi N, Pourmand MR (2019). Systemic infusion of autologous adipose tissue-derived mesenchymal stem cells in peritoneal dialysis patients: feasibility and safety. Cell J.

[58] Fan Y-P, Hsia C-C, Tseng K-W, Liao C-K, Fu T-W, Ko T-L (2016). The therapeutic potential of human umbilical mesenchymal stem cells from Wharton’s jelly in the treatment of rat peritoneal dialysis-induced fibrosis. Stem Cells Transl Med.

[59] Dymowska M, Aksamit A, Zielniok K, Kniotek M, Kaleta B, Roszczyk A (2021). Interaction between macrophages and human mesenchymal stromal cells derived from bone marrow and Wharton’s jelly: a comparative study. Pharmaceutics.

[60] Jiang H-Y, Wang J-P, Bai Y-H, Yang M, Zeng Y, Liao Y-J (2018). Clinical observation of umbilical cord mesenchymal stem cell transplantation for treating patients receiving peritoneal dialysis. Ital J Urol Nephrol.

[61] Wang Y, Lu X, He J, Zhao W (2015). Influence of erythropoietin on microvesicles derived from mesenchymal stem cells protecting renal function of chronic kidney disease. Stem Cell Res Ther.

[62] Liu B, Guan Q, Li J, da Roza G, Wang H, Du C (2017). Mesenchymal stroma cells in peritoneal dialysis effluents from patients. Hum Cell.

[63] Han BL, Zhou L, Guan Q, da Roza G, Wang H, Du C (2018). In vitro expansion and characterization of mesenchymal stromal cells from peritoneal dialysis effluent in a human protein medium. Stem Cells Int.

[64] Zhou L, Zong M, Guan Q, da Roza G, Wang H, Qi H (2019). Protection of the peritoneal membrane by peritoneal dialysis effluent-derived mesenchymal stromal cells in a rat model of chronic peritoneal dialysis. Stem Cells Int.

[65] Zhang D, Wei G, Li P, Zhou X, Zhang Y (2014). Urine-derived stem cells: a novel and versatile progenitor source for cell-based therapy and regenerative medicine. Genes Dis.

[66] Burdeyron P, Giraud S, Hauet T, Steichen C (2020). Urine-derived stem/progenitor cells: a focus on their characterization and potential. World J Stem Cells.

[67] Kang HS, Choi SH, Kim BS, Choi JY, Park G-B, Kwon TG (2015). Advanced properties of urine derived stem cells compared to adipose tissue derived stem cells in terms of cell proliferation, immune modulation and multi differentiation. J Korean Med Sci.

[68] Zhang C, George SK, Wu R, Thakker PU, Abolbashari M, Kim T-H (2020). Reno-protection of urine-derived stem cells in a chronic kidney disease rat model induced by renal ischemia and nephrotoxicity. Int J Biol Sci.

[69] Yang Q, Chen W, Han D, Zhang C, Xie Y, Sun X (2020). Intratunical injection of human urine-derived stem cells derived exosomes prevents fibrosis and improves erectile function in a rat model of Peyronie’s disease. Andrologia.

[70] Dong X, Zhang T, Liu Q, Zhu J, Zhao J, Li J (2016). Beneficial effects of urine-derived stem cells on fibrosis and apoptosis of myocardial, glomerular and bladder cells. Mol Cell Endocrinol.

[71] Shen J, Zheng J, Saxena R, Zhang C, Tang L (2015). Novel source of human hematopoietic stem cells from peritoneal dialysis effluents. Stem Cell Res.

[72] Zhou P, Wirthlin L, McGee J, Annett G, Nolta J (2009). Contribution of human hematopoietic stem cells to liver repair. Semin Immunopathol.

[73] Nikolits I, Nebel S, Egger D, Kreß S, Kasper C (2021). Towards physiologic culture approaches to improve standard cultivation of mesenchymal stem cells. Cells.

[74] Hu C, Wu Z, Li L (2020). Pre-treatments enhance the therapeutic effects of mesenchymal stem cells in liver diseases. J Cell Mol Med.

[75] Li M, Jiang Y, Hou Q, Zhao Y, Zhong L, Fu X (2022). Potential pre-activation strategies for improving therapeutic efficacy of mesenchymal stem cells: current status and future prospects. Stem Cell Res Ther.

[76] Yoshida K, Nakashima A, Doi S, Ueno T, Okubo T, Kawano K-I (2018). Serum-free medium enhances the immunosuppressive and antifibrotic abilities of mesenchymal stem cells utilized in experimental renal fibrosis. Stem Cells Transl Med.

[77] Romano B, Elangovan S, Erreni M, Sala E, Petti L, Kunderfranco P (2019). TNF-stimulated gene-6 is a key regulator in switching stemness and biological properties of mesenchymal stem cells. Stem Cells.

[78] Yu Y, Yoo SM, Park HH, Baek SY, Kim Y-J, Lee S (2019). Preconditioning with interleukin-1 beta and interferon-gamma enhances the efficacy of human umbilical cord blood-derived mesenchymal stem cells-based therapy via enhancing prostaglandin E2 secretion and indoleamine 2,3-dioxygenase activity in dextran sulfate sodium-induced colitis. J Tissue Eng Regen Med.

[79] Ezquerra S, Zuleta A, Arancibia R, Estay J, Aulestia F, Carrion F (2021). Functional properties of human-derived mesenchymal stem cell spheroids: a meta-analysis and systematic review. Stem Cells Int.

[80] Guo Y, Wang L, Gou R, Wang Y, Shi X, Zhang Y (2021). Ameliorative role of SIRT1 in peritoneal fibrosis: an in vivo and in vitro study. Cell Biosci.

[81] Zhao N, Li H, Yan Y, Jiang R, He X (2017). Mesenchymal stem cells overexpressing IL-35 effectively inhibit CD4+ T cell function. Cell Immunol.

[82] Guo H, Li B, Wang W, Zhao N, Gao H (2018). Mesenchymal stem cells overexpressing IL-35: a novel immunosuppressive strategy and therapeutic target for inducing transplant tolerance. Stem Cell Res Ther.

[83] Liu F, Xie J, Zhang X, Wu Z, Zhang S, Xue M (2020). Overexpressing TGF-β1 in mesenchymal stem cells attenuates organ dysfunction during CLP-induced septic mice by reducing macrophage-driven inflammation. Stem Cell Res Ther.

[84] Chen K-S, Wang C-H, Yen T-H, Chen J-R, Hung M-J, Lin C-Y (2010). Potential role of bone marrow-derived cells in the turnover of mesothelium. Ren Fail.

[85] Lee RH, Pulin AA, Seo MJ, Kota DJ, Ylostalo J, Larson BL (2009). Intravenous hMSCs improve myocardial infarction in mice because cells embolized in lung are activated to secrete the anti-inflammatory protein TSG-6. Cell Stem Cell.

[86] Eleuteri S, Fierabracci A (2019). Insights into the secretome of mesenchymal stem cells and its potential applications. Int J Mol Sci.

[87] Wang N, Li Q, Zhang L, Lin H, Hu J, Li D (2012). Mesenchymal stem cells attenuate peritoneal injury through secretion of TSG-6. PLoS ONE.

[88] Shi Y, Jiang N, Li M, Zeng X, Tian X (2023). Mesenchymal stem cells and connective tissue diseases: from bench to bedside. J Transl Intern Med.

[89] Koike Y, Li B, Lee C, Alganabi M, Zhu H, Chusilp S (2020). The intestinal injury caused by ischemia-reperfusion is attenuated by amniotic fluid stem cells via the release of tumor necrosis factor-stimulated gene 6 protein. FASEB J.

[90] Choi H, Lee RH, Bazhanov N, Oh JY, Prockop DJ (2011). Anti-inflammatory protein TSG-6 secreted by activated MSCs attenuates zymosan-induced mouse peritonitis by decreasing TLR2/NF-κB signaling in resident macrophages. Blood.

[91] Xiao GH, Jeffers M, Bellacosa A, Mitsuuchi Y, Vande Woude GF, Testa JR (2001). Anti-apoptotic signaling by hepatocyte growth factor/Met via the phosphatidylinositol 3-kinase/Akt and mitogen-activated protein kinase pathways. Proc Natl Acad Sci USA.

[92] Vasandan AB, Jahnavi S, Shashank C, Prasad P, Kumar A, Prasanna SJ (2016). Human Mesenchymal stem cells program macrophage plasticity by altering their metabolic status via a PGE2-dependent mechanism. Sci Rep.

[93] Németh K, Leelahavanichkul A, Yuen PST, Mayer B, Parmelee A, Doi K (2009). Bone marrow stromal cells attenuate sepsis via prostaglandin E2—dependent reprogramming of host macrophages to increase their interleukin-10 production. Nat Med.

[94] Liu F, Qiu H, Xue M, Zhang S, Zhang X, Xu J (2019). MSC-secreted TGF-β regulates lipopolysaccharide-stimulated macrophage M2-like polarization via the Akt/FoxO1 pathway. Stem Cell Res Ther.

[95] Yu M-A, Shin K-S, Kim JH, Kim Y-I, Chung SS, Park S-H (2009). HGF and BMP-7 ameliorate high glucose-induced epithelial-to-mesenchymal transition of peritoneal mesothelium. J Am Soc Nephrol JASN.

[96] Nakamura S, Niwa T (2005). Pyridoxal phosphate and hepatocyte growth factor prevent dialysate-induced peritoneal damage. J Am Soc Nephrol JASN.

[97] Khubutiya MS, Vagabov AV, Temnov AA, Sklifas AN (2014). Paracrine mechanisms of proliferative, anti-apoptotic and anti-inflammatory effects of mesenchymal stromal cells in models of acute organ injury. Cytotherapy.

[98] Qiu G, Zheng G, Ge M, Wang J, Huang R, Shu Q (2018). Mesenchymal stem cell-derived extracellular vesicles affect disease outcomes via transfer of microRNAs. Stem Cell Res Ther.

[99] Holm MM, Kaiser J, Schwab ME (2018). Extracellular vesicles: multimodal envoys in neural maintenance and repair. Trends Neurosci.

[100] Harrell CR, Fellabaum C, Jovicic N, Djonov V, Arsenijevic N, Volarevic V (2019). Molecular mechanisms responsible for therapeutic potential of mesenchymal stem cell-derived secretome. Cells.

[101] Zhao AG, Shah K, Cromer B, Sumer H (2020). Mesenchymal stem cell-derived extracellular vesicles and their therapeutic potential. Stem Cells Int.

[102] Mathieu M, Martin-Jaular L, Lavieu G, Théry C (2019). Specificities of secretion and uptake of exosomes and other extracellular vesicles for cell-to-cell communication. Nat Cell Biol.

[103] Leavitt RJ, Limoli CL, Baulch JE (2019). miRNA-based therapeutic potential of stem cell-derived extracellular vesicles: a safe cell-free treatment to ameliorate radiation-induced brain injury. Int J Radiat Biol.

[104] Mardpour S, Hamidieh AA, Taleahmad S, Sharifzad F, Taghikhani A, Baharvand H (2019). Interaction between mesenchymal stromal cell-derived extracellular vesicles and immune cells by distinct protein content. J Cell Physiol.

[105] Zhu S, Yao F, Qiu H, Zhang G, Xu H, Xu J (2018). Coupling factors and exosomal packaging microRNAs involved in the regulation of bone remodelling. Biol Rev Camb Philos Soc.

[106] Hu X, Shen N, Liu A, Wang W, Zhang L, Sui Z (2022). Bone marrow mesenchymal stem cell-derived exosomal miR-34c-5p ameliorates RIF by inhibiting the core fucosylation of multiple proteins. Mol Ther J Am Soc Gene Ther.

[107] Li Y, Shen Z, Jiang X, Wang Y, Yang Z, Mao Y (2022). Mouse mesenchymal stem cell-derived exosomal miR-466f-3p reverses EMT process through inhibiting AKT/GSK3β pathway via c-MET in radiation-induced lung injury. J Exp Clin Cancer Res CR.

[108] Baral H, Uchiyama A, Yokoyama Y, Sekiguchi A, Yamazaki S, Amalia SN (2021). Antifibrotic effects and mechanisms of mesenchymal stem cell-derived exosomes in a systemic sclerosis mouse model: possible contribution of miR-196b-5p. J Dermatol Sci.

[109] Exosomal lnc-CDHR derived from human umbilical cord mesenchymal stem cells attenuates peritoneal epithelial-mesenchymal transition through AKT/FOXO pathway Aging. 2023;15(14):6921–32. 10.18632/aging.v15i14. 10.18632/aging.204883 10.18632/aging.204883 PMC10415546 37466443

[110] Cruz-Barrera M, Flórez-Zapata N, Lemus-Diaz N, Medina C, Galindo C-C, González-Acero L-X (2020). Integrated analysis of transcriptome and secretome from umbilical cord mesenchymal stromal cells reveal new mechanisms for the modulation of inflammation and immune activation. Front Immunol.

[111] Shapouri-Moghaddam A, Mohammadian S, Vazini H, Taghadosi M, Esmaeili S-A, Mardani F (2018). Macrophage plasticity, polarization, and function in health and disease. J Cell Physiol.

[112] Li Q, Zheng M, Liu Y, Sun W, Shi J, Ni J (2018). A pathogenetic role for M1 macrophages in peritoneal dialysis-associated fibrosis. Mol Immunol.

[113] Stevens HY, Bowles AC, Yeago C, Roy K (2020). Molecular crosstalk between macrophages and mesenchymal stromal cells. Front Cell Dev Biol.

[114] Deng Y, Zhang Y, Ye L, Zhang T, Cheng J, Chen G (2016). Umbilical cord-derived mesenchymal stem cells instruct monocytes towards an IL10-producing phenotype by secreting IL6 and HGF. Sci Rep.

[115] Jin L, Deng Z, Zhang J, Yang C, Liu J, Han W (2019). Mesenchymal stem cells promote type 2 macrophage polarization to ameliorate the myocardial injury caused by diabetic cardiomyopathy. J Transl Med.

[116] Hyvärinen K, Holopainen M, Skirdenko V, Ruhanen H, Lehenkari P, Korhonen M (2018). Mesenchymal stromal cells and their extracellular vesicles enhance the anti-inflammatory phenotype of regulatory macrophages by downregulating the production of interleukin (IL)-23 and IL-22. Front Immunol.

[117] Song W-J, Li Q, Ryu M-O, Ahn J-O, Bhang DH, Jung YC (2018). TSG-6 released from intraperitoneally injected canine adipose tissue-derived mesenchymal stem cells ameliorate inflammatory bowel disease by inducing M2 macrophage switch in mice. Stem Cell Res Ther.

[118] Zhao Y, Zhu X-Y, Song T, Zhang L, Eirin A, Conley S (2021). Mesenchymal stem cells protect renal tubular cells via TSG-6 regulating macrophage function and phenotype switching. Am J Physiol Renal Physiol.

[119] Huang Q, Cheng X, Luo C, Yang S, Li S, Wang B (2021). Placental chorionic plate-derived mesenchymal stem cells ameliorate severe acute pancreatitis by regulating macrophage polarization via secreting TSG-6. Stem Cell Res Ther.

[120] Regmi S, Pathak S, Kim JO, Yong CS, Jeong J-H (2019). Mesenchymal stem cell therapy for the treatment of inflammatory diseases: challenges, opportunities, and future perspectives. Eur J Cell Biol.

[121] Yin S, Ji C, Wu P, Jin C, Qian H (2019). Human umbilical cord mesenchymal stem cells and exosomes: bioactive ways of tissue injury repair. Am J Transl Res.

[122] Alatab S, Najafi I, Atlasi R, Pourmand G, Tabatabaei-Malazy O, Ahmadbeigi N (2018). A systematic review of preclinical studies on therapeutic potential of stem cells or stem cells products in peritoneal fibrosis. Ital J Urol Nephrol.

[123] Qin T, Wu L, Hua Q, Song Z, Pan Y, Liu T (2020). Prediction of the mechanisms of action of Shenkang in chronic kidney disease: a network pharmacology study and experimental validation. J Ethnopharmacol.

[124] Jiang C, Lin W, Wang L, Lv Y, Song Y, Chen X (2020). Fushen granule, a traditional Chinese medicine, ameliorates intestinal mucosal dysfunction in peritoneal dialysis rat model by regulating p38MAPK signaling pathway. J Ethnopharmacol.

[125] He J, Wang M, Yang L, Xin H, Bian F, Jiang G (2022). Astragaloside IV alleviates intestinal barrier dysfunction via the AKT-GSK3β-β-catenin pathway in peritoneal dialysis. Front Pharmacol.

[126] Zhao J-L, Zhang T, Shao X, Zhu J-J, Guo M-Z (2019). Curcumin ameliorates peritoneal fibrosis via inhibition of transforming growth factor-activated kinase 1 (TAK1) pathway in a rat model of peritoneal dialysis. BMC Complement Altern Med.

[127] He H, Yang T, Li F, Zhang L, Ling X (2021). A novel study on the immunomodulatory effect of umbilical cord derived mesenchymal stem cells pretreated with traditional Chinese medicine Asarinin. Int Immunopharmacol.

[128] Xiong Y, Tang R, Xu J, Jiang W, Gong Z, Zhang L (2022). Tongxinluo-pretreated mesenchymal stem cells facilitate cardiac repair via exosomal transfer of miR-146a-5p targeting IRAK1/NF-κB p65 pathway. Stem Cell Res Ther.

[129] Baştuğ F, Gündüz Z, Tülpar S, Torun YA, Akgün H, Dörterler E (2014). Compare the effects of intravenous and intraperitoneal mesenchymal stem cell transplantation on ultrafiltration failure in a rat model of chronic peritoneal dialysis. Ren Fail.

